# Is Modular Dual Mobility Superior to Standard Bearings for Reducing Dislocation Risk after Primary Total Hip Arthroplasty? A Retrospective Comparative Multicenter Study

**DOI:** 10.3390/jcm12134200

**Published:** 2023-06-21

**Authors:** Vincenzo Ciriello, Roberta La China, Danilo Francesco Chirillo, Giuseppe Bianco, Federico Fusini, Ugo Scarlato, Carlo Albanese, Giancarlo Bonzanini, Lorenzo Banci, Lucio Piovani

**Affiliations:** 1 Ortopedia e Traumatologia, Ospedale Santa Croce e Carle, 12100 Cuneo, Italy; vincenzo.ciriello83@gmail.com (V.C.); piovani.l@ospedale.cuneo.it (L.P.); 2 Ortopedia e Traumatologia, Ospedale Santo Spirito, 15033 Casale Monferrato, Italy; dfchirillo@libero.it; 3 Ortopedia e Traumatologia, Ospedale Regina Montis Regalis, 12084 Mondovì, Italy; giuseppe.bianco@aslcn1.it (G.B.); fusinif@hotmail.com (F.F.); 4 Ortopedia e Traumatologia, Ospedale Civile, 10015 Ivrea, Italy; ugosca@libero.it (U.S.); carlo.albanese@edu.unito.it (C.A.); 5 Ortopedia e Traumatologia, Ospedale Sant’Antonio e Margherita, 15057 Tortona, Italy; gbonzanini@aslal.it; 6 Clinical Research Department, Permedica Orthopaedics, 23807 Merate, Italy; lorenzo.banci@permedica.it

**Keywords:** dislocation, intraprosthetic dislocation, modular dual mobility, standard bearing, total hip arthroplasty

## Abstract

Background: Dual mobility (DM) has been proven to reduce dislocation risk after total hip arthroplasty (THA). In the last decade modular DM (modDM) constructs have been introduced to allow the use of DM articulation with standard cementless acetabular shells. However, clinical evidence of modDM effectiveness is still low in primary THA and concerns about implant-related complications are increasing. This retrospective comparative multicenter study is aimed to investigate if the dislocation rate after primary THA could be reduced with modDM in comparison to standard bearing (SB). Methods: 262 THAs were performed between 2017 and 2019, using SB (129 hips) or modDM (133) with the same cementless highly porous modular acetabular cup. Dislocations, complications and revisions were recorded and implant survival was analyzed. Results: At 2.5-year mean follow-up, dislocation occurred in 4 hips (3.1%) within the SB group while intraprosthetic dislocation in 2 hips (1.5%) within the modDM group (*p* = 0.44). Implant survivals with revision due to dislocation were 95.2% and 95.9% at 4-year follow-up for SB and modDM, respectively (*p* = 0.50). Conclusions: modDM used in primary THA might reduce dislocation rate in comparison to SB, even in high-risk patients, however, caution is advocated due to specific intraprosthetic dislocation.

## 1. Introduction

Dislocation remains a major complication following primary total hip arthroplasty (THA), occurring from 0.4% to 0.7% after elective THA [1,2] and from 1.6% to 6% after THA for femoral neck fracture (FNF) [1,3].

The dual mobility (DM) acetabular articulation was first introduced in the 1970s to reduce dislocation risk after THA. The DM concept was developed by combining the benefit of a large femoral head, which increases joint stability by higher jumping distance, with the benefit of a small diameter femoral head which reduces polyethylene wear [4,5]. Conventional DM acetabular components consist of a monobloc cobalt-chromium (CoCr), or stainless steel, an acetabular shell that articulates with a mobile polyethylene retentive liner (large articulation) coupled with a 28 mm, or 22 mm, diameter ceramic or metal femoral head (small articulation).

DM THA for elective primary elective procedures and for FNF showed favorable results with lower dislocation rates than unipolar THA [6,7].

In the last decade, modular DM (modDM) constructs have been introduced to allow the use of DM articulation with standard cementless titanium acetabular shells in primary and revision THA [8,9]. The modDM construct consists of a modular CoCr, or stainless steel, a liner that is seated into a standard titanium acetabular shell to allow articulation with conventional DM bearings. Several brands of modDM acetabular liners are available today on the market [10].

Advantages of modDM include the possibility to use a standard press-fit acetabular cup, the use of additional fixation screws for further cup stabilization, the ability to check cup seating by looking through cup screw holes, and to intraoperatively switch from a standard fixed liner to a DM articulation in case of soft tissues poor tensioning. Nowadays, the market offers a large portfolio of similar modDM designs from different orthopedic companies.

The use of DM and modDM cups in primary and revision THA is rapidly growing in clinical practice, aiming to reduce dislocation and revision in high-risk patients such as those with FNF [11].

However, the use of modDM is not free from complications. Recently, a growing number of studies in the literature have raised concerns regarding the use of this type of implant. Dislocation rate after modDM THA is reported to range from 0.07% to 1.17% after primary procedures and even higher after revision surgeries [12,13]. During the last decade, intraprosthetic dislocation (IPD) has been reported as a specific failure mode of DM cups which can occur after both conventional DM THA [14] and modDM THA at early or late stage [15,16,17].

The present study aimed to investigate if the use of modDM in primary THA is justified by a significant reduction in dislocation rate in comparison to unipolar primary THA with standard bearing (SB) implanted with the same cementless acetabular cup.

## 2. Material and Methods

### 2.1. Study Design

This observational study is a retrospective data collection from a multicentric consecutive series of primary THA performed in five hospitals of the Piemonte Region, Italy. The coordinator study site was Santa Croce e Carle Hospital at Cuneo. Other study sites were Santo Spirito Hospital at Casale Monferrato, Civile Hospital at Ivrea, Sant’Antonio e Margherita Hospital at Tortona and Regina Montis Regalis Hospital at Mondovì. All study sites were tertiary care public hospitals with a large number of trauma procedures besides elective surgery, where a highly-porous acetabular cup, which allows to use of a polyethylene or ceramic SB or a modDM, was introduced in clinical practice in 2017.

This study was approved on June 2022 by the Ethics Committee of Cuneo with protocol number 112-2022. The study was conducted in accordance with the ethical standards laid down in the 1964 Declaration of Helsinki and its later amendments. All patients enrolled in the study gave their written informed consent.

Inclusion criteria were primary cementless total hip arthroplasty performed using the same highly-porous acetabular component between 2017 and 2019, with an SB or modDM articulation and minimum 2-year follow-up. No particular exclusion criteria were used as the study was observational.

The primary endpoint of the study was the number of dislocations that occurred in the group of THAs with modDM compared to the group of THAs with SB. Further endpoints were any other complication, surgical reintervention, revision, and clinical and radiographic outcomes in both groups.

### 2.2. Prosthetic Device

**Figure 1:**
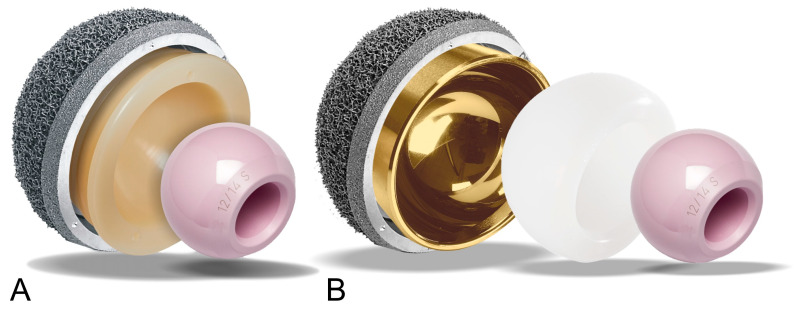
Image of the cementless acetabular cup implanted in all included patients, the Jump System Traser^®^ acetabular cup by Permedica Orthopaedics, Merate, Italy. Traser^®^ is a 3D-printed cancellous bone-like irregular titanium lattice, additively manufactured by selective laser melting in one-step process together with the cup. Traser^®^ has 70% fully interconnected porosity with a mean pore size of 520 microns. The Jump System Traser^®^ cup allows for seating of a standard fixed insert, as vitamin E blended cross-linked polyethylene (**A**), or a modular stainless steel DM insert, fully coated by titanium-niobium nitride (**B**).

All hips included in the study received the same cementless press-fit acetabular cup, the Jump System Traser^®^ cup by Permedica Orthopaedics, Merate, Italy (Figure 1).

The cup features on the bone interface a cancellous bone-like highly porous titanium structure (Traser^®^), which is 3D-printed by selective laser melting with Ti6Al4V powder for additive manufacturing. The acetabular cup was implanted using, according to the surgeon’s intraoperative assessment, standard unipolar bearings (SB) as ceramic (Biolox Delta^®^, CeramTec, Plochingen, Germany) or vitamin E-blended moderately cross-linked polyethylene (VitalXE^®^) liners coupled with 32 or 36 mm femoral heads, or a modDM construct (Figure 1). The modular DM option was mainly indicated for high-risk patients for instability as patients with a femoral neck fracture, advanced age, abductors atrophy and complex primary THA.

Surgical procedures were performed by five experienced senior surgeons (L.P., U.S., G.B., G.B., D.C.) and their junior trainees. Each senior surgeon performs usually more than 100 hip arthroplasties per year. A posterior lateral surgical approach was used in all procedures with joint capsule repair when possible. Press-fit of the acetabular shell was achieved thanks to the polar flattened profile of the cup and the high friction of its trabecular structure against bone.

The acetabulum was prepared by reaming at least one further millimeter than the selected size of the acetabular cup, allowing for optimal cup positioning. Otherwise, a line-to-line preparation of the acetabulum would have led to incomplete cup seating and equatorial press-fit.

### 2.3. Study Procedure

Through a hospital database search, all primary THA procedures performed in the study centers from January 2017 to December 2019 with the Jump System Traser^®^ cup were identified.

Surgery registers were evaluated to collect information on implant components and on any intraoperative complications. This study was conducted by examining medical records, outpatient reports, and radiographic images of the selected patients according to inclusion criteria. Dislocations and IPDs were assessed and registered. IPDs were classified as early or late IPDs if occurred within 24 months after surgery or later, respectively [15].

### 2.4. Clinical Evaluation

Clinically, patients were evaluated by the Harris Hip Score (HHS) [18] and assessed preoperatively and postoperatively at each follow-up.

### 2.5. Radiographic Analysis

Radiographic analysis was performed on the more recently available anteroposterior and lateral radiographs of the hips taken at the latest patient follow-up and compared with the first postoperative anteroposterior and lateral radiographs. Radiographic signs of interest were cup malpositioning defined as inclination ≥ 55° and/or anteversion ≥ 40° [19,20], periprosthetic fractures, heterotopic ossification classified according to Brooker [21], the presence of a polar gap not completely filled by new bone behind the cup, periprosthetic radiolucency lines and osteolysis in the areas described by DeLee e Charnley [22], osseointegration signs according to Moore [23], cup loosening defined as >3 mm of cup migration from radiographic landmarks as the line drawn between the acetabular fossae or Kohler lines, or >4° of cup rotation from the initial cup inclination measured as the angle formed between the inter teardrop line and the line drawn through the cranial and caudal equatorial edges of the cup [24].

### 2.6. Statistical Analysis

Statistical analysis was performed using GraphPad Prism 8.0 statistical software (San Diego, CA, USA). Continuous variables were reported as mean ± standard deviation and dichotomous variables were reported as numbers and percentages.

Analysis of continuous variables was performed with the t-Student test for unpaired samples, whereas analysis of dichotomous variables with the Chi-square test, or Fisher Exact test when at least one variable resulted ≤5. Statistical significance was set with a *p*-value < 0.05.

The Mann–Whitney test was used to analyze differences between non-parametric variables. Implant cumulative probability of survival was determined according to the Kaplan–Meier method with a 95% confidence interval with revision of any components due to dislocation as the end-point. Lost to follow-up cases were included as censored cases in the survival analysis. Log-rank test was performed to test the difference in survival rate between the study groups. Logistic regression analysis was performed to identify which covariates related to patient and implant in the study cohort were able to influence the risk of postoperative dislocation, which was considered as the dependent variable. Hips were categorized into those which dislocated at least once versus those which not, without considering subsequent revision. Univariate and multivariate analyses were conducted for demographic, implant, and surgical independent variables which were considered for describing study groups. Results were reported as odds ratios, *p*-value and 95% confidence interval.

## 3. Results

Overall, 262 primary THAs were included with an average follow-up of 2.5 years (range 0.2–4.1). 129 hips received standard polyethylene (83), or ceramic (46) liners (SB group) and 133 hips received modDM (modDM group). The study population and implant data are summarized in Table 1.

At the last follow-up, 13 patients (13 hips) died due to reasons unrelated to hip arthroplasty, 1 patient (1 hip) was lost to follow-up and 5 patients (5 hips) were surgically reoperated with revision of the acetabular liners. No acetabular cup was revised in the whole cohort. All acetabular cups were found to be radiographically stable and well osseointegrated, with slight radiolucency in 9 hips (3.4%) in zone C1 (2), C2 (5) and C3 (4).

HHS improved from 51.9 preoperatively to 91.9 postoperatively in the SB group at the latest available follow-up and from 45.1 to 90.7 in the modDM group. A significant difference was found between SB and modDM in preoperative HHS but not postoperatively.

**Figure 2:**
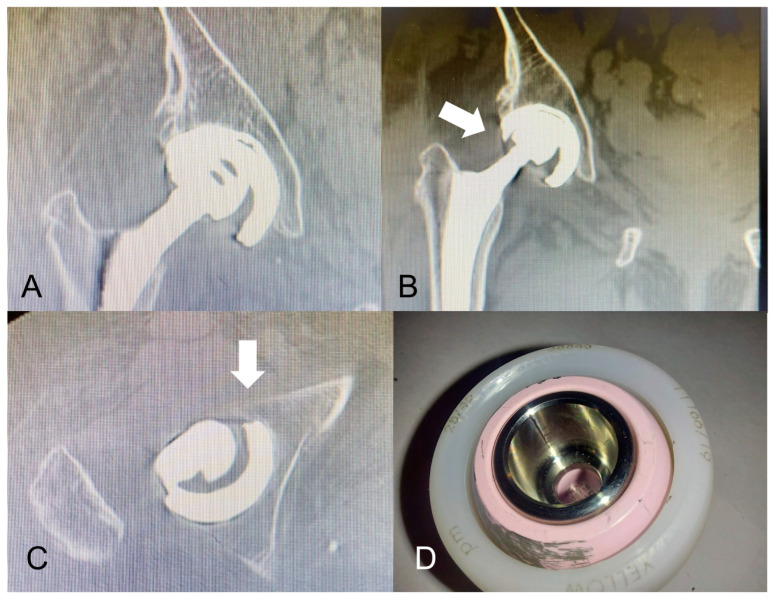
Early IPD of a modular DM construct occurred 11 months after revision surgery of a THA with a standard fixed polyethylene insert and 32 mm femoral head which previously failed for dislocation at a 19-month follow-up. CT scan (images (**A**–**C**)) was taken before re-revision. (**A**): CT scan image shows the eccentric position of the small femoral head with respect to the acetabular shell. It is well recognized the modular adapter between the ceramic head and the femoral stem morse taper. (**B**): the modular CoCr liner appears to be malseating into the titanium acetabular shell: the superior-lateral margin of the metal liner (white arrow) is more prominent from the acetabular shell equatorial rim than its medial margin. Another radiographic sign of modular liner malseating is the eccentric void space between the liner and shell. (**C**): white arrow shows how the acetabular cup was placed with excessive anteversion and modular CoCr liner malseating. (**D**): wear signs on the retrieved ceramic head surface due to its articulation against the modular CoCr liner edge.

In the SB group, the dislocation occurred postoperatively in four patients (4 hips, 3.1%), of which three required surgical reintervention with replacement of the acetabular liner, while one was treated by closed hip reduction. In particular, one of these dislocations occurred in a 71-year-old female patient, 19 months after the index THA and was treated by replacing the polyethylene liner with a modDM construct using a modular CoCr insert coupled with a dual mobility polyethylene liner and a 28 mm ceramic femoral head assembled over the femoral stem by means of a titanium XL sleeve adaptor. Afterward, 11 months later the revision surgery, another dislocation occurred with IPD. At the re-revision surgery, the acetabular shell was left in situ as it was stable and well osseointegrated, but the ceramic head which was dislocated, and the mobile polyethylene liner which showed visible wear signs, were both replaced with a second modDM construct (Figure 2).

**Figure 3:**
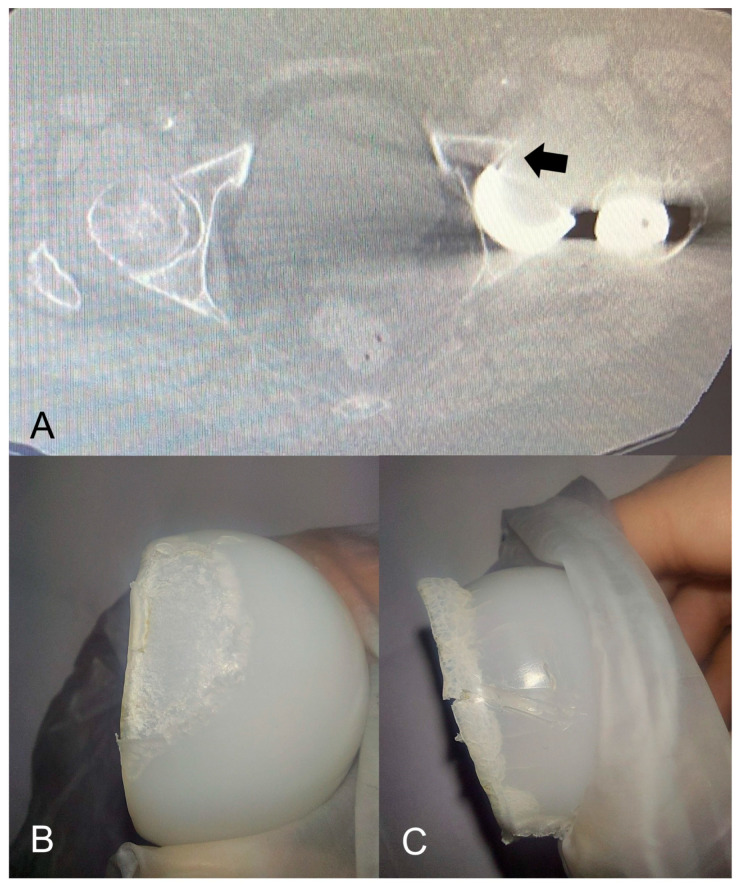
IPD case occurred 41 months after modular DM THA. (**A**): CT scan image showing the acetabular cup implanted with 55° of anteversion, leaving the anterior portion of the acetabular cavity uncovered (black arrow). (**B**,**C**): Massive wear and deformation of the mobile polyethylene liner were observed at revision along the liner edge. These signs on the retrieved component could represent the catastrophic results of a mobile liner impingement against the anterior portion of the acetabulum caused by cup malpositioning for excessive anteversion.

In the modDM group, IPD occurred in two patients (1.5%). One IPD occurred in a 78-year-old female patient with osteoarthritis and severe obesity (BMI 37.5) at a 41-month follow-up after modDM THA. Closed reduction was unsuccessfully attempted and revision surgery was needed to replace the 28 mm ceramic head and the mobile polyethylene insert which were intraoperatively found dissociated and massively damaged (Figure 3).

**Figure 4:**
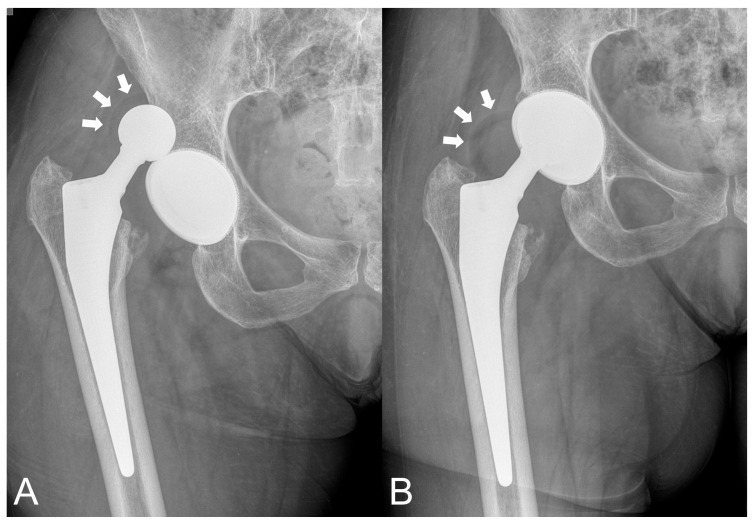
Early IPD case occurred 2 months after modular DM THA during unsuccessful hip closed reduction to treat a dislocation caused by a traumatic event. (**A**): Plain radiograph taken after dislocation, showing outside articulation the mobile polyethylene liner correctly assembled over the 28 mm ceramic head (white arrows). (**B**): Plain radiograph taken immediately after the hip closed reduction attempt, showing the IPD with the mobile polyethylene liner dissociated from the small head, the so-called “bubble sign” (white arrows). Polyethylene liner dissociation is likely due to the great traction forces during the closed reduction maneuver which are higher and overwhelm the push out force of the retentive mobile polyethylene liner.

The second IPD occurred in a 66-year-old female patient during the attempt of hip closed reduction for a dislocation caused by a traumatic event two months after the modDM THA performed for FNF (Figure 4).

**Figure 5:**
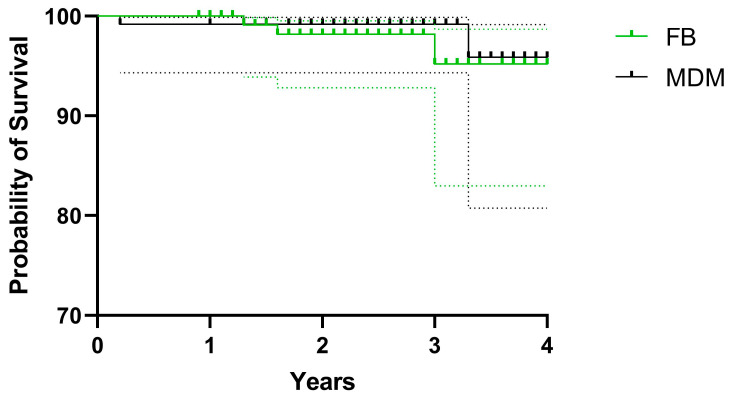
Kaplan–Meier survival of standard bearing and modular DM groups with revision of any component due to dislocation as the end-point.

Implant cumulative survival with revision of any component, due to dislocation as the endpoint, was 95.2% [95% CI, 82.9–98.7%] and 95.9% [95% CI, 80.8–99.2%] at 4-year follow-up for SB and modDM groups, respectively (*p* = 0.5019) (Figure 5).

All other complications are summarized in Table 2.

Logistic regression analysis was performed to identify the independent covariates reported in Table 1 which were able to bias dislocation rate. The multivariate analysis was performed including those covariates which resulted significantly different in Table 1, or significant in the univariate analysis. Results are reported in Table 3 as odds ratio (OR) and 95% confidence interval (CI). Cup malpositioning resulted to have significant OR from the univariate analysis but not from the multivariate analysis.

**Figure 6:**
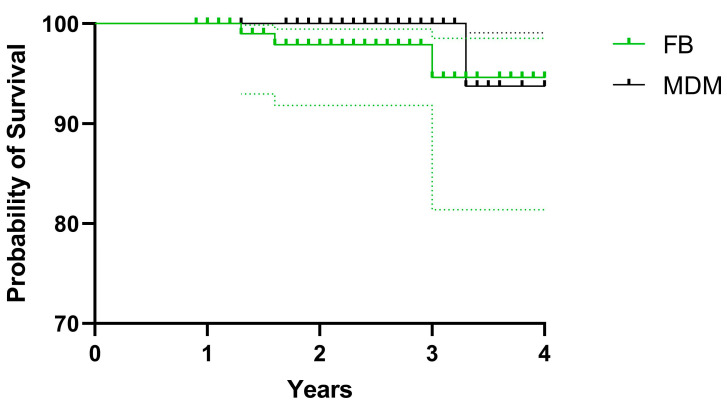
Stratified analysis (excluding hips with femoral neck fractures as index diagnosis) for Kaplan Meier survival of standard bearing and modular DM subgroups with revision of any component due to dislocation as the end-point.

Since FNF, as the index diagnosis, resulted in unequal distribution between the two groups, a stratified analysis for dislocation and implant survival has been performed, excluding from the analysis those hips with FNF, because, even if not found as a significant covariate in the regression analysis, FNF is well known to be a risk factor for dislocation after THA [3]. Again, implant survival analysis with revision of any component, due to dislocation as the endpoint, gave comparable results, with 94.62% [81.37–98.52%] vs. 93.75% [63.24–99.09%] at 4-year follow-up for SB and modDM subgroups, respectively (*p* = 0.3979) (Figure 6).

Complications and revisions in both subgroups are summarized in Table 4. The dislocation rate in SB and modDM resulted from 3.5% vs. 1.1%, again with no significant difference.

## 4. Discussion

The results of the present study suggest that the use of modDM in primary THA might ensure a lower risk of dislocation in comparison to SB, facing the lower dislocation rate observed (1.5% vs. 3.1%), although no statistical significance was found. This dislocation rate reduction is confirmed by the stratified analysis (1.1% vs. 3.5%, respectively) which shows a higher difference, still not statistically significant, but with significant clinical relevance. The reasons for the lack of statistical significance may rely on the non-homogeneous and small-sized groups. In fact, modDM has been used mainly in patients with a high risk of instability. The dislocation rate in modDM has been likely influenced by confounding factors such as cup malpositioning or femoral neck fracture, even though the multivariate regression analysis did not show any significant covariate predictive for dislocation. Similar results are reported by Singh et al. [25] who conducted a retrospective propensity score-matched analysis between SB vs monobloc DM vs modular DM. The authors found no statistical difference in the 90-day all-cause revision rate (3.4% vs. 2.7% vs. 0.7%; *p* = 0.265) and the 90-day revision rate due to dislocation (1.3% vs. 0.7% vs. 0.0%; *p* = 0.365) between the SB, modular DM, and monobloc DM cohorts, respectively.

Dislocation and failure rates associated with the use of modDM in primary THA seem to be low as seen in the recent literature. Excellent results were recently reported up to 3-year follow-up, with only one dislocation out of a pooled series of 636 (0.15%) MDM implants by Stryker from three retrospective studies [8,26,27]. A systematic review with meta-analysis reported a prevalence of 0.6% and 0.8% for dislocation and intraprosthetic dislocation, respectively, after primary THA was performed with modern DM implants [28].

Good results were also shown with modDM in revision surgery and complex primary THA [29,30], even if a recent study reported discordant results with higher than expected dislocation rate (11%) after revision THA with modDM, with three IPDs [31].

In our study, we recorded two IPD cases within the modDM group (1.5%). The first case was an early IPD that occurred during an attempt at hip closed reduction for a traumatic dislocation which occurred at a 2-month follow-up. In this patient, the cup was well positioned (Figure 4). The second case was a late IPD which was very atypical and did not occur during the hip reduction maneuver. In fact, 41 months of follow-up for this IPD is too short a period of time after THA for expecting usual wear at the polyethylene retentive rim caused by impingement against the prosthetic femoral neck (third joint wear). At revision surgery, massive wear with deformation of the mobile liner was observed along the liner edge, and metal wear signs on the ceramic head were observed (Figure 3). The extensive damage on the retrieved polyethylene liner, as well as the excessive cup anteversion, noticed on CT scans, have contributed to a mobile liner malfunction with impingement against the uncovered bone of the acetabulum anterior portion left uncovered by the malpositioned cup (Figure 3B).

Moreover, a further IPD case occurred in our study after the revision of a unipolar SB implant which was converted to a modDM, and, even if this case was not within the modDM group, it was anyway described and discussed hereafter as considered of interest. This third IPD case was not recorded within the modDM group but occurred in one revised patient of the SB group, who experienced a first dislocation 19 months after unipolar THA, which needed a revision THA by replacing the standard polyethylene liner with a modDM construct. This IPD occurred 11-months after the first revision. The reason which might have caused this early IPD may be multifactorial including initial cup malpositioning in excessive anteversion, small cup size (Ø 48 mm), malseating of the modular CoCr liner (visible on CT scans, Figure 2B,C) and the use of an extra-long femoral head adapter (Figure 2A,D). Acetabular cup sizes of ≤50 mm are known to be at higher risk for modDM liner malseating [32], as well as for non-optimal stability [33].

Thus, the IPD rate found in the present study regarding modDM implants (1.5% and 1.1%, if including or excluding FNF, respectively) can be considered comparable with other IPD and dislocation rates published up to date.

First described by Lecuire et al. [34] in 2004, IPD is the dissociation between the small femoral head and the polyethylene mobile liner of a DM articulation due to loss of its retentive mechanism and this complication can occur postoperatively at any time [15]. IPD may have different etiologies such as high-energy trauma, the attempt of close hip reduction after dislocation, off-label use of DM components, poor compression force for DM component intraoperative assembly, acetabular cup malposition and polyethylene wear [15,35].

IPD was defined as late IPD or early IPD, according to the time of its occurrence. Early IPD can occur within 24 months after index surgery, while late IPD occurs afterward. While late IPD is a wear-related, mid- to long-term complication specific to all DM bearings, usually caused by the impingement between the neck of the femoral stem and the rim of the polyethylene liner, early IPD has different etiologies [15]. De Martino et al. [15] reported that almost 50% of early IPD episodes occurred with modDM constructs, of which the majority (15 out of 19 early IPDs) is caused by an attempt of closed hip reduction. Addona et al. [16], from the same group and center (Hospital for Special Surgery, NY), reported a high prevalence of early IPD after closed hip reduction for dislocation with 5 cases out of 154 DM and modular DM implants (3.2%) which led to revision surgery to either another DM bearing or constrained liner. However, early IPD can happen also spontaneously for other reasons such as poor impaction of the polyethylene insert over the femoral head, cup malpositioning, polyethylene wear due to impingement with femoral neck and off-label use of modDM liners [15,36].

In our study, all two IPDs with modDM were associated with small cup sizes, while three out of four dislocations in the SB group with greater cup sizes. Moreover, the IPD case found after the revision for dislocation in the SB group was similarly associated with modDM liner malseating in 48 mm-sized acetabular cups. We cannot confirm that malseating could have had a role in the etiology of the IPD, but certainly, the use of modDM associated with small component sizes, especially for revision surgery, might require particular care by surgeons. In fact, modDM has been addressed to be biomechanically unfavorable in terms of hip stability in comparison to standard DM or SB [33,37].

Acetabular cup size ≤ Ø 50 mm has been identified as a risk factor for liner malseating and dislocation after the use of modDM constructs [31,32]. Malseating rate of modDM liners after THA is currently reported to range from 1.2% [13,38] to 5.8% [32,39] with no clinical consequence but likely with a predisposition to increased fretting corrosion [39].

Furthermore, findings of the present study showed how, in our daily clinical practice, modDM is mainly used in patients undergoing THA for FNF or osteonecrosis in comparison to unipolar SBs, which instead are mostly preferred in younger patients with osteoarthritis. Moreover, the use of modDM is associated with elderly female patients older than 70 years, with a history of osteoporosis or poor bone quality, and with lower preoperative HHS. The higher number of cups within the modDM group implanted with the use of additional fixation screws confirmed this trend. The use of modDM in these specific patients confirms its advantage to allow a DM articulation in more fragile patients at higher risk of dislocation, whilst maintaining the advantage of the fixation screw option of standard press-fit acetabular cups in case of poor bone quality. Another observed trend, even if not statistically significant, is the preferred use of modDM when the acetabular cup is positioned suboptimally.

In our study, we did not find any significant differences between SB and modDM groups regarding the number of hips which presented with periarticular HO. Conversely, to Rashid et al. [40], who found a higher prevalence of HO with DM THA in comparison to standard THA in patients with FNF, in this study, we found instead a higher prevalence of HO in SB THA, even if not statistically significant, in comparison to modDM THA.

The present study had some limitations due to its own design. Since the study population was a retrospective cohort and the data collection observational, the resulting groups were not homogeneous in terms of demographic and implant parameters, particularly regarding patient diagnoses. To address this study drawback, we performed first a logistic regression analysis to identify which patient and implant factors were able to influence dislocation risk, then we performed a stratified analysis for dislocation rate and implant survival excluding those hips affected by FNF, as this is considered a main bias for dislocation [3]. A propensity score matching to avoid covariate bias should be the best method for group matching, but it would have substantially reduced the sample size of the available patients with the same kind of implant. The study sample size, which was too small, may have probably influenced the significance of the results. Being a retrospective observational study, a proper sample size calculation was not performed as the sample size was already established by the available number of THAs performed in the considered period. Another limitation in the study method involved the acetabular cup positioning that was not radiographically measured by inclination and anteversion angles but malpositioning was only assessed as a dichotomous variable.

## 5. Conclusions

Modular DM implants, even being a viable option for reducing dislocation risk in patients with a higher risk of instability, are however susceptible to specific early and late IPD risk in comparison to unipolar SB implants. Findings from this study suggested that modDM used in primary THA might reduce dislocation rate even in high-risk patients in comparison to SB, although no statistical significance was found. In conclusion, modDM constructs should be used in primary THA with caution only in high-risk patients for dislocation, keeping in mind that dislocation and IPD can still occur with this type of implant, leading to survival rates for dislocation comparable to SB.

## Figures and Tables

**Table 1:** Demographic and implant data of the study population.

Group	Standard Bearing	Modular DM	*p*-Value
N. of hips	129	133	
Mean follow-up (SD; range)	2.4 (0.7; 0.9–4.1)	2.6 (0.7; 0.2–4.1)	0.0247
Diagnosis, N. of hips			
Osteoarthritis	93	60	0.0001
Dysplasia	9	8	0.7520
Osteonecrosis	7	22	0.0041
Rheumatoid arthritis	3	2	0.6815
Femoral neck fracture	17	41	0.0006
Gender (male:female)	54:75	39:94	0.0340
Mean age (SD; range)	67.1 (9.5; 26–87)	75.6 (7.6; 54–94)	0.0001
Mean BMI (SD; range)	25.8 (3.0; 21–39)	24.7 (3.6; 21–42)	0.0002
Mean preop. HHS (SD; range)	51.9 (16.1; 3–81)	45.1 (18.3; 0–86)	0.0049
N. of hips with history of osteoporosis or poor bone quality	22	44	0.0028
Mean acetabular cup size, mm (SD; range)	52.6 (3.5; 46–60)	51.0 (3.6; 44–62)	0.0003
N. of acetabular cups with additional fixation screws (mean N. of screws)	13 (1.7)	27 (2.1)	0.0214
N. of hips with cup malpositioning	1	6	0.1203

**Table 2:** Dislocations, revisions and other complications occurred in standard bearing and modular DM groups. * All dislocations associated with modular DM were IPD. CI, confidence interval.

Group	Standard Bearing	Modular DM	*p*-Value
Hips	129	133	
N. of hips with dislocation *	4 (3.1%)	2 (1.5%)	0.4416
Femoral head size (mm) of dislocation cases	36, 36, 32, 32	28, 28	
Acetabular cup size (mm) of dislocation cases	60, 54, 52, 48	50, 48	0.0557
N. of acetabular insert revisions for dislocation (any reason)	3 (2.3%)	2 (1.5%)	0.6804
Implant survival rate with revision of any component due to dislocation [95% CI]	95.17% [82.98–98.69%]	95.87% [80.77–99.16%]	0.5019
Other complications	2 groin pain 1 spe deficit 3 superficial infections 1 intraop acetabular fracture 1 intraop femoral fracture 14 HO	1 pulmonary embolism 2 superficial infections 1 intraop acetabular fracture 1 intraop femoral fracture 1 bone fracture for trauma 8 HO	

**Table 3:** Univariate and multivariate logistic regression analysis of patient and implant factors for postoperative dislocation after index total hip arthroplasty. OR Odds ratio, CI Confidence interval. Bold type is used for values when statistical significance has been found.

Patient/Implant Factors	Univariate Analysis				Multivariate Analysis			
	OR	*p*-Value	95% CI		OR	*p*-Value	95% CI	
Osteoarthritis	1.43	0.67	0.27	10.49	0.23	0.41	0.01	8.86
Femoral neck fracture	0.69	0.74	0.03	4.44	2.13	0.67	0.03	81.95
Dysplasia	3.00	0.32	0.15	20.13	-	-	-	-
Gender (male)	0.90	0.91	0.12	4.73	-	-	-	-
Age	1.00	0.96	0.92	1.10	1.03	0.70	0.88	1.28
BMI	1.15	0.21	0.89	1.41	1.24	0.15	0.91	1.74
Preop. HHS	0.99	0.78	0.95	1.04	0.97	0.46	0.89	1.04
History of osteoporosis or poor bone quality	0.58	0.63	0.03	3.73	-	-	-	-
Acetabular cup size	1.01	0.91	0.79	1.25	0.92	0.74	0.52	1.36
Cup malpositioning	**25.10**	**0.001**	3.00	166.3	7.58	0.26	0.15	336.7
Group (modDM)	0.47	0.39	0.06	2.48	2.07	0.66	0.04	83.63

**Table 4:** Stratified analysis for dislocation and other complications between standard bearing group and modular DM group, selecting only hips with all types of diagnosis excluding femoral neck fractures. THA for Femoral neck fractures is known to be at higher risk of dislocation.

Group	Standard Bearing	Modular DM	*p*-Value
Hips	112	92	
N dislocations	4 (3.5%)	1 (1.1%)	0.3810
N. of acetabular insert revisions for any reason	3 (2.7%)	1 (1.1%)	0.6286
Implant survival rate with revision of any component due to dislocation [95% CI]	94.62% [81.37–98.52%]	93.75% [63.24–99.09%]	0.3979
Complications	2 groin pain 3 superficial infections 1 intraop acetabular fracture 1 intraop femoral fracture 8 HO	2 superficial infections 1 intraop acetabular fracture 1 bone fracture for trauma 5 HO	

## Data Availability

Data available on request and not publicly available due to restrictions of data protection and privacy. The data presented in this study are available on request from the corresponding author and with permission of “Ospedale Santa Croce e Carle, Cuneo”.
